# Brief History of Early Lithium-Battery Development

**DOI:** 10.3390/ma13081884

**Published:** 2020-04-17

**Authors:** Mogalahalli V. Reddy, Alain Mauger, Christian M. Julien, Andrea Paolella, Karim Zaghib

**Affiliations:** 1 Centre of Excellence in Transportation Electrification and Energy Storage (CETEES), Hydro-Québec, 1806, Lionel-Boulet blvd., Varennes, QC J3X 1S1, Canada; MogalahalliVenkatesh.VenkatashamyReddy@hydro.qc.ca (M.V.R.); paolella.andrea@hydro.qc.ca (A.P.); 2 Institut de Minéralogie, de Physique des Matériaux et de Cosmochimie (IMPMC), Sorbonne Université, UMR-CNRS 7590, 4 Place Jussieu, 75005 Paris, France; alain.mauger@upmc.fr (A.M.); christian.julien@upmc.fr (C.M.J.)

**Keywords:** intercalation compounds, lithium batteries, electrolyte, cathode, anode

## Abstract

Lithium batteries are electrochemical devices that are widely used as power sources. This history of their development focuses on the original development of lithium-ion batteries. In particular, we highlight the contributions of Professor Michel Armand related to the electrodes and electrolytes for lithium-ion batteries.

## 1. Introduction

Lithium “lithion/lithina” was discovered in 1817 by Arfwedson [1] and Berzelius [2] by analyzing petalite ore (LiAlSi_4_O_10_), but the element was isolated through the electrolysis of a lithium oxide by Brande and Davy in 1821 [3]. It was only a century later that Lewis [4] began exploring its electrochemical properties. Considering lithium’s excellent physical properties, such as its low density (0.534 g cm^−3^), high specific capacity (3860 mAh g^−1^), and low redox potential (−3.04 V vs. SHE), it was quickly realized that lithium could serve well as a battery anode. 

In early 1958, Harris [5] examined the solubility of lithium in various non-aqueous (aprotic) electrolytes—including cyclic esters (carbonates, γ-butyrolactone, and γ-valerolactone), molten salts, and inorganic lithium salt (LiClO_4_)—dissolved in propylene carbonate (PC). He observed the formation of a passivation layer that was capable of preventing a direct chemical reaction between lithium and the electrolyte while still allowing for ionic transport across it, which led to studies on the stability of lithium-ion batteries [5,6]. These studies also increased interest in the commercialization of primary lithium-ion batteries. 

Since the late 1960s, non-aqueous 3 V lithium-ion primary batteries have been available in the market with cathodes including lithium sulfur dioxide Li//SO_2_ in 1969 [7]; lithium–polycarbon monofluoride (Li//(CF_x_)_n_ commercialized by Matsushita in 1973; lithium–manganese oxide (Li//MnO_2_) batteries commercialized by Sanyo company in 1975, initially sold in solar rechargeable calculators (Sanyo, Lithium Battery Calculator, Model CS-8176L); lithium–copper oxide (Li//CuO) batteries still used today [8]; and (Li/LiI/Li_2_PVP) batteries with an Li metal anode, a lithium iodine electrolyte, and a polyphase cathode of polyvinyl-pyridine (PVP) used in cardiac pacemakers since 1972 (see Holmes [9] for the history of this primary battery). Simultaneously, advances in the understanding of the intercalation of lithium in different materials gave birth to rechargeable (secondary) lithium-ion batteries. In this review, we report a brief history of these secondary batteries that have now taken an important place in our daily life, as we find them in many devices ranging from portable phones to electric vehicles. Attention is focused on the beginning of their development in the period of 1970–1990. The contribution of Michel Armand is highlighted in this context.

## 2. Intercalation Cathode Development

In the early 1970s, research was rekindled in the area of the intercalation reactions of an ion, atom, or molecule into a crystal lattice of a host material without destroying the crystal structure. The following general criteria are needed for reversible intercalation reactions: (a) the materials must be crystalline; (b) there must be empty sites in the host crystal lattice in the form of isolated vacancies or as one-dimensional (1D) channels, 2D layers (van der Waals gap), or channels in a 3D network; and (c) both electronic and ionic conductivity must be present for reversible Li intercalation–deintercalation [10,11]. Based on these criteria, pioneering intercalation studies on Prussian-blue materials, such as iron cyanide bronzes M_0.5_Fe(CN)_3_, were demonstrated by Armand et al. [12] in 1972. The same year, the topic of energy storage devices and the concept of solid-solution electrodes and electrolyte components for lithium-based secondary batteries were discussed at a NATO conference in Italy, where Brian Steele suggested the use of transition metal disulfides as intercalation electrode materials [13]. Other groups, notably Gamble et al. [14] and Dines et al. [15,16] (EXXON, USA), evaluated transition-metal chalcogenide (*M*S_2_, with *M* = Ta, Nb, and Ti) electrode materials. At the same conference, Armand suggested the use of several inorganic materials and transition metal oxides, reported the use of CrO_3_ within graphitic planes as an electrode material for both Li and Na batteries, and described the first solid-state battery using β-alumina as a solid electrolyte [17].

In 1974, inspired by the pioneering work of Rao et al. [18] (IBM, USA) and the group of Rouxel [19] (Nantes, France) who demonstrated the fast kinetics of the intercalation reactions in metal disulfides, Whittingham patented the Li//TiS_2_ battery [20,21], and the electrochemical properties of Li^0^//TiS_2_ battery were simultaneously investigated by Whittingham [22] and Winn et al. [23,24] in 1976. One decade later, Li//TiS_2_ cells were commercialized under the form of standard-sized XR2016 coin cells by Eveready Battery Co., USA for CMOS memory back-up applications [25,26], under an AA-sized form by Grace Co., USA with a capacity of 1 Ah [27] and under a C-sized form for a cell operating at temperature in the range of −20 to +20 °C with a capacity of 1.6 Ah [28]. Another metal disulfide, namely MoS_2_, also met success: Li^0^//MoS_2_ cells (MOLICEL^TM^) were manufactured by Moli Energy Ltd. in Canada, with an energy density of 60–65 Wh kg^−1^ at a discharge rate of C/3 (800 mA) [29]. Among other metal chalcogenides investigated at that time, only NbSe_3_ emerged. Following the study of this material as a cathode element by Murphy et al. [30] in 1976, AT&T (USA) commercialized an AA-sized Li^0^//NbSe_3_ cylindrical cell operating over 200 cycles at a current of 400 mA with a capacity of 0.7 Ah in 1989 [31]. For completeness, we mention V_2_O_5_, which was also used as a cathode element of commercialized lithium-ion batteries, but this was only done in the 1990s. Since the present review focuses on the early development of lithium batteries, we guide the reader to a book for further details concerning them [32].

Inspired by the studies on Na_x_CoO_2_ by the group of Hagenmuller [33] in 1973, Goodenough et al. [34] replaced Na with Li and proposed LiCoO_2_ as a new cathode (3.9 V vs. Li^+^/Li) that they patented in 1979. LiCoO_2_ is more stable in air than NaCoO_2_, and its good electrochemical properties [35] have earned it the most commercialized cathode for decades. This result opened up a new approach for research into the development of solid-solution materials, in particular Li(Ni_x_Mn_y_Co_z_)O_2_ (NMC) in the 1990s, as reviewed elsewhere [32].

Early work on spinel LiMn_2_O_4_ was carried out in 1984 by Thackeray et al. [36]. Mn is low cost compared to Co, and the thermal stability of LiMn_2_O_4_ is better than that of LiCoO_2_. The problem of Mn’s dissolution into electrolytes at high temperature, however, was not solved until Zhou et al. found an effective salt, LiFNFSI, which improves resilience [37].

Further advances were made in the development of olivine-based cathodes, and in particular LiFePO_4_, which was pioneered by the Goodenough’s group [38]. The watershed for the use of these materials was the discovery of a carbon-coating process discovered in an international lab (France-Québec) directed by Armand (see Ref. [39] and references therein). 

LiFePO_4_ has a remarkable thermal stability, but its redox potential (3.5 V vs. Li^+^/Li) is small. LiCoO_2_ has a rather poor stability, but it belongs to the class of 4 V cathodes. Therefore, in parallel to the development of the LiFePO_4_ battery, further research was done to improve the thermal stability of LiCoO_2_ by the synthesis of solid solutions involving doping by Ni, Mn, and non-transition elements. Early work in 1992 by the group of Delmas et al. [40] suggested a solid-solution concept that was then explored by many groups to optimize the Li(Ni_x_Mn_y_Co_z_)O_2_ (NMC) cathode that is now commercialized by various companies due to its high energy density; it now shares the market with LiFePO_4_.

## 3. Development of Anode Materials

In addition to the development of positive (cathode) electrode materials, research was also carried out on Li-metal and Li-alloy negative (anode) electrodes. Early batteries were commercialized with such anodes [25,26,27,28,29,30,31]. However, they faced safety concerns due to the formation of anode dendrites. 

The insertion of lithium in graphite dates from 1955, but this was only confirmed by the synthesis of LiC_6_ in 1965 [41]. The synthesis of LiC_6_, however, was not obtained by electrochemical process at that time, and the reversible intercalation of lithium in graphite up to LiC_6_ was established by Besenhard and Eichinger in 1976 [42,43], but, owing to a lack of a suitable electrolyte that could prevent co-intercalation at that time, graphite was not used as a cathode material. This problem was solved by the group of Armand in 1978 by the use of polymer electrolyte that allowed these authors to identify the suitability of graphite as an intercalated negative electrode [44,45].

In the 1970s, Armand proposed the fabrication of a lithium-ion battery based on two different intercalation materials for both cathodes and anodes; this battery was named the rocking-chair battery (later the lithium-ion battery) due to the shuttle of ions from one electrode to another during the charge–discharge process [46]. This concept involved lithium ions being transferred from one side to the other [45] and was demonstrated in 1980 by Lazzari and Scrosati [47].

The rocking-chair battery concept was thus offered the same year as the LiCoO_2_ positive electrode was proposed by Goodenough, but the laboratory experiment has to be taken to the industrial scale. This was achieved by the commercialization of the LiCoO_2_//hard-carbon battery by the Sony and Asahi Kasei teams led by Nishi in 1991. The rocking-chair concept later gained major success in the Japanese battery industry with Sony [48] (1985) and Sanyo in 1988 [49].

In the 1990s, another anode material, the spinel Li_4_Ti_5_O_12_, was proposed for Li-ion batteries [50,51] and, more recently, for Na-ion batteries [52]. This anode may substitute graphite only when high-power density is needed, but in Na-ion batteries, the Li_4_Ti_5_O_12_ electrode delivers a reversible capacity of 155 mAh g^−1^ and presents the best cycle ability among all reported oxide-based anode materials [53].

## 4. Electrolytes 

Armand was a pioneer in the development of a polymer electrolytes based on polyethylene oxide-lithium salts (PEO:Li) [54,55]. Solid-state batteries have the advantage that they use Li as the anode the collector current. As a result, the theoretical energy density of solid-state batteries is larger than that of Li-ion batteries that use liquid electrolytes. The drawback is that the PEO and polymers in general have a poor ionic conductivity at room temperature, so that the commercial all-solid-state lithium-ion batteries that use polymers, such as the batteries in Bluecars^®^ and Bluebuses^®^, must be used at elevated temperatures. On another hand, liquid electrolytes are very good ionic conductors, and it is difficult to avoid the formation of dendrites at the surface of lithium metal. As a consequence, liquid electrolytes have been adopted in commercial batteries. Such is the case, for instance, for the above-mentioned LiCoO_2_ battery of Sony that used LiPF_6_ in propylene carbonate and diethyl carbonate (PC:DEC, 1:1) as the electrolyte. However, PC and DEC are not compatible with lithium metal, so Li-ion batteries with liquid electrolytes adopt the rocking-chair concept with graphite anodes proposed by Armand, implying a smaller theoretical energy density but a higher rate capability at room temperature than all-solid-state batteries. The use of graphite, however, implies that PC was commonly used in the first batteries, as its intercalation results in exfoliation of the carbon sheets. Indeed, the Sony LiCoO*_2_*-type battery used hard carbon as the anode. The long cycle life of Li-ion batteries with graphitic carbon and liquid electrolytes (without PC) was demonstrated by Basu [56] in 1981, who used a two-molar solution of LiAsF_6_ dissolved in 1,3 dioxolane. Only in the 1990s, however, did commercialized batteries emerge with a graphite anode using a liquid electrolyte with LiPF_6_ in carbonate solvents; this is still the standard today. 

In 1991, the group of Armand reported a novel salt: lithium bis(trifluoromethanesulfonyl)imide (LiTFSI) [57], now commonly used as an Li-ion conducting electrolytes for Li-ion batteries and later on a new class of single-ion solid polymer [58,59] and solvent-in-salt electrolytes [60], which gives more evidence that Armand was one of the researchers that played a major role in the development of lithium-ion batteries in the period of time covered in this review. Actually, the period of time where he played a major role is continuing. Further details, including the more recent contributions of Armand to the field of electrolytes, were discussed in a recent review [46], but his recent contributions include advanced materials in all components, including electrodes, of lithium- and sodium-ion batteries [61,62].

Prior patents that paved the route to the present Li-ion batteries are reported in Table 1, and the increase of the energy density of the batteries through the early years of rechargeable batteries is illustrated in Table 2. Note, however, that this figure can only give a partial view of the progress since energy density is not the only parameter that is meaningful. For instance, a battery with an LiFePO_4_ cathode and a Li_4_Ti_5_O_12_ anode can be cycled over 30,000 cycles at very fast rate of 15C (4 min) and a discharge rate of 5C (12 min) [63]. This performance gives interest to such a battery for some applications, even though its energy density is smaller than that of the LiFePO_4_-graphite battery, since the operating voltage of the battery is reduced by 1.5 V. 

**Figure 1:**
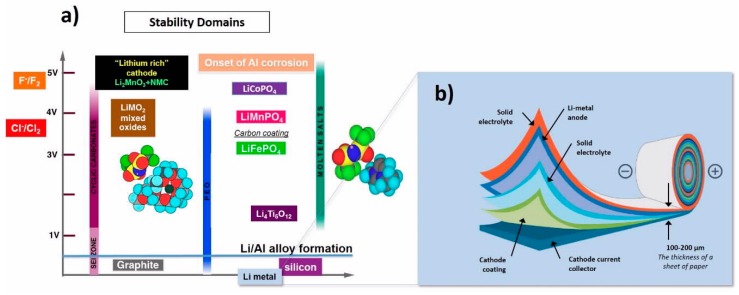
(**a**) Stability of different electrolytes and (**b**) solid state lithium metal polymer battery architecture proposed by Professor Michel Armand.

The stability of different electrolytes and the Li-polymer cell architecture proposed by Professor Michel Armand are illustrated in Figure 1. The sequence of stability ranges in the order cyclic carbonates (SEI zone) < polyethylene oxide (PEO) < molten salts.

## 5. Separators

For completeness, we mention the separator, which is the last important component of lithium-ion batteries. This element, however, has raised much less trouble than electrodes and electrolytes. As soon as 1970, a time where lithium metal was used as the anode of primary batteries with organic electrolytes, prototype models of Li//CuS cells had been developed by SAFT in France in a pilot line type of operation [64,65,66], using a non-woven polypropylene separator. The Li//V_2_O_5_ system patented by Livingston Electronic Corporation (now Honeywell) [6] and P. R. Mallory and Co. Inc. [67,68] used a cellulosic separator, e.g., filter paper or a Celgard^®^ separator of porous polypropylene. Another example is the Li//SO_2_ system [69]. Actually, most of the lithium metal batteries developed in the early 1970s already used a non-woven polypropylene separator. The alternative was a glass-fiber paper separator, like in the case of the Li//SOCl_2_ cell [70].

## 6. Conclusions

Fundamental works on lithium-ion batteries date from the 1970s, and remarkable progress has been made since the 1980s. The first commercial lithium-ion battery was issued in 1991, making it a rather short period of time between work in laboratories and the industrial production. In this review, we reported the main steps that led to this success. Among the people that contributed to this success from this beginning up to now, Michel Armand has played a key role in the creation and development of lithium-ion battery cathodes, anodes, and electrolytes. We deem him to be one of the “forefathers of modern batteries,” inspiring many academic and industrial researchers to design alternative electrode and electrolyte materials with high energy densities, long cycle lives, and low costs, leading to the further development of the batteries for electric vehicles and for the regulation of the current produced by intermittent sources before integration into smart grids. 

## Figures and Tables

**Table 1:** List of some of patents related to the early lithium-ion batteries.

Inventor/Company	Patent Title	Patent Number	Application Date
Armand, M.; Duclot, M. (ANVAR, France)	See Reference [44]	French 7,832,976	22 Nov. 1978
Goodenough, J.B.; Mizushima, K. (UK Atomic Energy Establishment)	Fast ion conductors (A_x_M_y_O_2_)	U.S. 4,357,215A	5 April 1979
Goodenough, J.B.; Mizushima, K. (UK Atomic Energy Establishment)	Electrochemical cell with new fast ion conductors	U.S. 4,302,518	31 March 1980
Basu, S. (Bell Labs Inc., USA)	Graphite/Li in molten salt	U.S. 4,304,825	21 Nov. 1980
Armand, M.; Duclot, M. (ANVAR, France)	See Reference [43]	U.S. 4,303,748	12 Jan. 1981
Ikeda, H.; Narukawa, K.; Nakashima, H. (Sanyo Co., Japan)	Graphite/Li in nonaqueous solvents	Japanese 1,769,661	18 June 1981
Basu S. (Bell Labs Inc., USA)	Graphite/Li in nonaqueous solvents	U.S. 4,423,125A	13 Sept. 1982
Yoshino, A.; Jitsuchika, K.; Nakajima, T. (Asahi Chemical Ind., Japan)	Li-ion battery based on carbonaceous material	Japanese 1,989,293	5 Oct. 1985
Nishi N., Azuma H., Omaru A. (Sony Corporation)	Non aqueous electrolyte cell	U.S. 4,959,281	29 Aug. 1989
Fujimoto, M.; Yoshinaga, N.; Ueno, K. (Japan)	Li-ion secondary batteries	Japanese 3,229,635	Nov. 1991

**Table 2:** Table of the main early rechargeable lithium batteries that were commercialized before 1991. Note that they all have a lithium metal anode, with the first lithium-ion battery with a carbon anode dating to 1991 and the rocking chair concept (Michel Armand) dating to 1970.

Electrochemical System	Voltage (V)	Specific Energy		Commercial Co. (Issue)
		Wh/kg	Wh/L	
Li//TiS_2_	2.1	130	280	Exxon (1978)
Li//LiAlCl_4_-SO_2_	3.2	63	208	Duracell (1981)
Li//NbSe_3_	2.0	95	250	Bell Telephone Lab. Inc. (1983)
LiAl//polyaniline	3.0	-	180	Bridgestone (1987)
Li//MoS_2_	1.8	52	140	MoLi Energy (1987)
Li//V_2_O_5_	1.5	10	40	Toshiba (1989)
LiAl//polypyrolle	3.0	-	180	Kanebo (1989)
Li//Li_0.3_MnO_2_	3.0	50	140	Tadiran (1989)
LiVO_x_	3.2	200	300	Hydro-Québec (1990)
C//LiCoO_2_	3.6	150–190	-	Sony (1991)
